# Effect of Coronavirus Disease 2019 Pandemic and Vaccination on Menstrual Cycle Among King Saud University Students in Riyadh: A Retrospective Online Survey

**DOI:** 10.7759/cureus.70277

**Published:** 2024-09-26

**Authors:** Kawther N Almosa, Sulaiman A Alshammari, Shahad M Almutairi, Yasmeen M Almousa

**Affiliations:** 1 Family and Community Medicine, King Saud University Medical City, Riyadh, SAU; 2 Family and Community Medicine, College of Medicine, King Saud University, Riyadh, SAU; 3 Internal Medicine, King Saud University Medical City, Riyadh, SAU; 4 Primary Health Care, Ministry of Health, Riyadh, SAU

**Keywords:** covid-19 pandemic, covid-19 vaccine, female students, menstrual cycle changes, psychologic stress

## Abstract

Objectives

We aimed to evaluate the prevalence of menstrual cycle changes following the COVID-19 pandemic and the COVID-19 vaccination among female students at King Saud University in Riyadh, Saudi Arabia.

Methods

A cross-sectional retrospective study using an online questionnaire was conducted between October 2022 and December 2022. Data were collected concerning demographic variables, clinical conditions, menstrual cycle characteristics, COVID-19 infection status, and vaccination history.

Results

Of the 525 female students who participated in the study (mean age, 21.5 ± 3.9 years; mean body mass index, 22.7 ± 4.7 kg/m^2^), 246 (46.9%) had tested positive for COVID-19, 15 (2.9%) had visited the emergency room, and eight (1.5%) had been hospitalized. The average duration between menstrual cycles increased significantly among the study sample (pre-pandemic, 25.9 ± 5.3 days; post-pandemic, 26.8 ± 6.7 days; p = 0.016). Pre-pandemic, 221 (42.1%) participants reported less than two days of variance between their shortest and longest cycles, as compared with 175 (33.3%) post-pandemic (p < 0.001). Regarding vaccination, 393 (74.9%) participants had received three vaccinations, and 110 (21%) had received two vaccinations. Post-vaccination, 184 (35%) participants reported no changes to their menstrual cycle, 154 (29.3%) reported less disruption, and 44 (8.4%) experienced more disrupted menstrual cycles.

Conclusion

This study highlights potential associations between the COVID-19 pandemic, subsequent vaccinations, and changes in menstrual patterns, which can help to elucidate the physiological and psychological effects of pandemics and vaccination campaigns on women’s health. Future studies must consider biological and psychosocial factors to elucidate underlying mechanisms and causal relationships.

## Introduction

The menstrual cycle is crucial to female health and well-being [1]. Since the COVID-19 pandemic outbreak, some have questioned the effects of vaccination on menstrual cycles and female reproductive health, resulting in a degree of public hesitancy toward vaccination [2]. Studies concerning the direct effects of vaccination on the menstrual cycle are limited. In a typhoid vaccine-related study conducted in 1913 in New York, >50% of participants who had received a typhoid vaccine reported menstrual irregularities, including missed, late, and early menstruation, discomfort, and excessive bleeding. These irregularities were transient, resolving after six months [3]. A 1982 study in Japan reported an association between hepatitis B vaccination and menstrual cycle disturbances [4]. More recently, a 2015 post-market safety study in Nagoya reported menstrual irregularities in >25% of recipients of the human papillomavirus vaccine [5]. In a retrospective online survey in the United Kingdom (UK) in 2021, 20% of vaccinated women experienced menstrual irregularities, with oral contraceptives (OCs) considered to have acted as a protective factor [6]. In the United States (US), a prospective cohort study of 2,403 vaccinated women indicated that COVID-19 vaccinations had altered the menstrual cycle duration but not the length of menses [7]. Another US study reported that 42% of women with regular menstrual cycles bled more than usual after vaccination, whereas 44% reported no difference [8]. A study in the Middle East and North Africa region published in 2022 found that 66.3% of participants experienced menstrual cycle changes after COVID-19 infection and vaccination [9]. One systematic review highlighted that many women reported changes in their menstrual cycles during the COVID-19 pandemic, which were attributed either to pandemic-related stress and behavioral changes or to illness due to COVID-19 infection itself [2]. A study undertaken in Jordan reported that prior to the pandemic, 17.5% of women experienced menstrual irregularities; however, while curfew was enforced, this percentage dropped to 10.5% (p = 0.01) [10]. A cross-sectional study in Saudi Arabia reported an association between menstrual cycle changes and the severity of COVID-19 infection, noting that these changes persisted in 6.4% of women for more than six months [11]. These study findings suggest that the COVID-19 pandemic and COVID-19 infection affected menstrual cycles either through stress or directly through illness. However, studies undertaken in Saudi Arabia concerning this issue are limited. Therefore, we undertook a retrospective study to determine the association between the COVID-19 pandemic and COVID-19 vaccination as well as menstrual cycle changes. Understanding this issue is crucial for counseling women in the study region and addressing vaccination hesitancy.

## Materials and methods

Following the STROBE Guidelines for Observational Studies, this study used a retrospective cross-sectional online survey conducted among female students at King Saud University (KSU) in Riyadh, Saudi Arabia. Postgraduate and undergraduate students participated in the study from October 2022 to December 2022. The survey questionnaire was distributed via email with the assistance of KSU female student affairs and the Student Council at KSU College of Medicine. Additionally, surveys were conducted on the KSU women’s campus. Inclusion criteria comprised KSU students of childbearing age who had been vaccinated against COVID-19. Pregnant students were excluded from this study.

Sample size calculation

The sample size was calculated to be 344 participants using the following formula:

 n = (z)^2^p(1 − p)/d^2^,

Where n is the sample size, Z is the Z-value (95% confidence level, 1.96), p is the prevalence of menstrual changes related to the COVID-19 pandemic/vaccination (34%), and d is the study precision (5%).

Questionnaire design

The questionnaire was designed based on relevant literature [7-9] and included the following study variables: (i) demographic variables: age, level of education, and academic pathway; (ii) clinical history: history of infection, history of chronic diseases, use of oral contraception, and body mass index (BMI); (iii) menstrual cycle characteristics; and (iv) history of COVID-19 infection and vaccination.

Validity and pilot testing

The questionnaire’s face and content validity were refined based on feedback from five experts (three professors of Family Medicine and two professors of Obstetrics and Gynecology) and participants. The survey was piloted on 28 individuals to test the questions’ applicability, clarity, and simplicity. The average time to complete the questionnaire ranged from 5 to 10 minutes.

Ethical considerations

The aims and objectives of the study were communicated to the participants. All demographic data were kept anonymous and confidential and used solely for research. The study followed the principles of the Declaration of Helsinki. It was approved by the Institutional Research Board Committee (IRB) of the Health Sciences Colleges Research on Human Subjects at KSU (IRB Approval No. E-22-7140). All participants gave informed consent at the beginning of the questionnaire.

Data analysis

Data were analyzed using IBM SPSS Statistics for Windows, version 26.0 (IBM Corp., Armonk, NY, USA) software. Descriptive statistics, including means, standard deviations (SDs), frequencies, and percentages, were used to analyze changes in the participants’ menstrual cycles. The paired t-test was used to compare the means of cycle lengths and the number of days of bleeding before and after the onset of the COVID-19 pandemic. The chi-squared test was used to evaluate differences in the observed frequency distribution across categories, in this case, menstrual cycle irregularity before and after the pandemic. Statistical significance was set at a p-level of 0.05 (95% confidence interval [CI]).

## Results

In total, 525 female students from KSU in Riyadh participated in this study. The mean age of the participants was 21.5 ± 3.9 years, and the mean BMI was 22.7 ± 4.7 kg/m^2^. Most participants, 274 (52.2%) students, were studying medicine. The baseline characteristics of the recruited female students are presented in Table 1.

**Table 1:** Baseline socio-demographics of the sample population (N = 525) M, mean; SD, standard deviation; BMI, body mass index

Variable	n (%)
Age (M ± SD)	21.5 ± 3.9
Weight (M ± SD)	58.1 ± 12.6
Height (M ± SD)	159.0 ± 12.2
BMI (M ± SD)	22.7 ± 4.7
Educational attainment	
Undergraduate	502 (95.6%)
Postgraduate	23 (4.4%)
What discipline are you studying?	
Medicine	274 (52.2%)
Science	147 (28%)
Humanities	94 (17.9%)
Administration	10 (1.9%)

The COVID-19-related characteristics of the students are presented in Table 2. In all, 246 (46.9%) participants received a positive COVID-19 test result, and 15 (2.9%) visited an emergency department. Post-infection diagnoses included post-viral fatigue syndrome, thyroid dysfunction, and other conditions, whereas pre-pandemic diagnoses included endometriosis, polycystic ovary syndrome, uterine polyps, eating disorders, and thyroid disorders. Pre-pandemic, 183 (34.9%) of the participants reported heavy periods. In the year following the pandemic, 129 (24.6%) participants reported lighter periods, 121 (23%) reported no change, 53 (10.1%) reported heavier periods, and 179 (34.1%) reported both lighter and heavier periods. The severity of pre-menstrual syndrome among the participants was rated at 5.8 out of 10 on average. Symptom changes during menstruation varied, with 82 (34.7%) reporting no changes.

**Table 2:** COVID-19-related characteristics of the sample population (N = 525) M, mean; SD, standard deviation

Item	n (%)
Did you have a positive COVID-19 test?	
Yes	246 (46.9%)
No	279 (53.1%)
Were you hospitalized?	
Yes	8 (1.5%)
No	474 (90.3%)
I visited the Accident and Emergency Department	15 (2.9%)
Urgent care but was not admitted	8 (1.5%)
Did not stay overnight at the hospital	1 (0.2%)
I was not hospitalized, but I think I should have been	19 (3.6%)
Since your infection with COVID-19, have you been formally diagnosed with any of the following?	
None	468 (89.1%)
Post-viral fatigue syndrome	5 (1%)
Thyroid dysfunction	7 (1.3%)
Other	45 (8.6%)
Before the beginning of the COVID-19 pandemic, had you been formally diagnosed with any of the following conditions?	
Endometriosis	4 (0.8%)
Polycystic ovary syndrome (PCOS)	26 (5%)
Uterine polyps	2 (0.4%)
Uterine fibroids	0 (0%)
Eating disorders	6 (1.1%)
Thyroid disorders	10 (1.9%)
None	477 (90.8%)
Before the beginning of the COVID-19 pandemic, would you say your periods were particularly heavy?	
Yes	183 (34.9%)
No	642 (65.1%)
Before the beginning of the COVID-19 pandemic, on a scale from 0 (not at all) to 10 (a lot), how much did you suffer from premenstrual syndrome (PMS)? (M ± SD)	5.1 ± 2.8
Over the last year, following the pandemic, have you noticed any changes in your periods?	
Lighter	129 (24.6%)
No changes	121 (23%)
Heavier	53 (10.1%)
Lighter and heavier	179 (34.1%)
I don’t know	43 (8.2%)
Over the last year, on a scale from 0 (not at all) to 10 (a lot), how much did you suffer from premenstrual syndrome (PMS)? (M ± SD)	5.8 ± 2.8
Over the last year, following the pandemic, have you noticed any changes in symptoms DURING menstruation?	
Less	49 (9.3%)
No changes	182 (34.7%)
More	209 (39.8%)
I don’t know	85 (16.2%)

Table 3 presents the menstrual cycle characteristics of the recruited female students before and after the COVID-19 pandemic. The average duration between cycles, from the start of one bleed to the beginning of the next, showed a significant increase from 25.9 ± 5.3 days pre-pandemic to 26.8 ± 6.7 days post-pandemic (p = 0.016). The regularity of menstrual cycles significantly changed during the pandemic (p < 0.001). Pre-pandemic, 221 (42.1%) participants reported a variance of more than two days between their shortest and longest cycles. Post-pandemic, 175 (33.3%) reported the same level of regularity. The proportion of students who experienced a cycle length difference of two to five days increased from 166 (31.6%) pre-pandemic to 171 (32.6%) post-pandemic. In contrast, those who experienced a cycle length difference of >20 days decreased from 48 (9.1%) to 38 (7.8%). Lastly, the average duration of the menstrual period, defined as the number of days of bleeding, slightly decreased from 5.9 ± 1.8 days pre-pandemic to 5.7 ± 1.9 days after the onset of the pandemic. However, this difference was not statistically significant (p = 0.08).

**Table 3:** Menstrual cycle characteristics among the study sample pre- and post-COVID-19 pandemic (N = 525)

Item	n (%)	n (%)	p-value
	Pre-pandemic	Post-pandemic	
Before the beginning of the COVID-19 pandemic, how many days long, on average, was your cycle (duration between the start of one bleed and the start of the next bleed)? (M ± SD)	25.9 ± 5.3	26.8 ± 6.7	0.016
Before the beginning of the COVID-19 pandemic, how irregular were your menstrual cycles?			
Less than two-day difference between the shortest and longest cycles	221 (42.1%)	3.3%)	0.0001
2- to 5-day difference between the shortest and longest cycles	166 (31.6%)	-32.60%	
5- to 10-day difference between the shortest and longest cycles	11.8%)	-18.90%	
10- to 20-day difference between the shortest and longest cycles	28 (5.3%)	6.9%)	
>20-day difference between the shortest and longest cycles	48 (9.1%)	38 (7.8%)	
I did not have periods	0 (0%)	6 (1.1%)	
Before the beginning of the COVID-19 pandemic, how many days long, on average, was your period (number of days of bleeding)? (M ± SD)	5.9 ± 1.8	5.7 ± 1.9	0.08

Table 4 presents the experiences of the recruited female students related to menstrual cycle and COVID-19 vaccination. Most participants, 393 (74.9%), had received three vaccine doses. Post-vaccination changes in menstrual cycles were reported as follows: 184 (35%) participants noted no changes, whereas 198 (37.7%) stated that their menstrual cycles were disrupted. In all, 480 (91.4%) participants did not use hormonal contraceptives, and 458 (87.1%) had no chronic illnesses. Of the participants who reported using OCs, pills were the most common type. Anti-inflammatory medications were the most commonly used non-contraceptive medication.

**Table 4:** Experiences of study sample related to menstrual cycle and COVID-19 vaccination (N = 525)

Item	n (%)
Have you been vaccinated against COVID-19?	
Yes, one dose	19 (3.6%)
Yes, two doses	110 (21%)
Yes, three doses	393 (74.9%)
No	3 (0.6%)
Have you noticed any changes to your menstrual cycles since you got vaccinated?	
No	184 (35%)
Yes, my menstrual cycles are LESS disrupted	154 (29.3%)
Yes, my menstrual cycles are MORE disrupted	44 (8.4%)
May be	143 (27.2%)
If yes, what happened to your cycle exactly? (You can choose more than one)	
Longer period days	36 (6.9%)
Shorter period days	65 (12.4%)
Heavier period	44 (8.4%)
Lighter period	53 (10.1%)
Irregular	97 (18.5%)
No change	230 (43.8%)

Table 5 describes using OCs and other medications among the study sample during the pandemic. Most participants did not use hormonal contraceptives (472, 89.9%) or had no chronic illnesses (458, 87.1%). Of the participants who reported using hormonal contraceptives, OCs were the most common type (39, 7.4%), and most had been using OCs for <12 months (10, 1.9%). Most participants did not regularly use medications (416, 79.2%), with anti-inflammatory medicines being the most common (45, 8.6%) among those who did. Lastly, when receiving vaccinations, only 16 (3%) participants took OCs, whereas most were not (509, 97%).

**Table 5:** Use of hormonal contraception and medications among study sample (N = 525)

Variables	n (%)
In the past 12 months, did you take any contraceptives?	
Yes	45 (8.6%)
No	480 (91.4%)
If yes, which type?	
Injections	1 (0.2%)
Intrauterine device	2 (0.4%)
Oral contraceptive pills	39 (7.4%)
Patches	3 (0.6%)
Other	28 (5.3%)
How long have you been using hormonal contraceptives continuously (without an interruption of more than 1 month)?	
None	472 (89.9%)
Less than 6 months	23 (4.4%)
Less than 1 year	10 (1.9%)
1-2 years	15 (2.9%)
2-3 years	0 (0%)
3-5 years	0 (0%)
5-10 years	0 (0%)
For 10 years or more	5 (1%)
Over the last year, have you regularly been using any of the following medications?	
No, I am not taking any regular medications	416 (79.2%)
Anti-inflammatory medications such as ibuprofen or aspirin	45 (8.6%)
Antidepressants (SSRIs, e.g., Cipramil, Priligy, Cipralex, Prozac, Faverin, Seroxat, Lustral, and Brintellix)	23 (4.4%)
Other	41 (7.8%)
Do you have any chronic illnesses?	
No chronic illnesses	458 (87.1%)
Diabetes mellitus	9 (1.7%)
Dyslipidemia	9 (1.7%)
Bronchial asthma	35 (6.7%)
Polycystic ovary syndrome	40 (7.6%)
Were you on oral contraceptive pills while taking the vaccine?	
Yes	16 (3%)
No	509 (97%)

## Discussion

Our findings concerning menstrual cycle changes related to the COVID-19 pandemic and vaccinations of female students at KSU in Riyadh provide valuable insights into potential physical changes linked to the pandemic and subsequent vaccination campaigns. Most of the participants in our study were undergraduate medical students, indicating that the sample was predominantly composed of younger women at the beginning of their academic journeys. This group, primarily engaged in medical education, may face unique stressors that could influence their health. Our findings suggest that many women experienced menstrual changes following the COVID-19 pandemic and subsequent vaccination. This result is consistent with a study conducted in the UK by Alvergne et al., which surveyed a large cohort of women across various reproductive ages to investigate the prevalence and risk factors of menstrual changes following COVID-19 vaccination [12]. The UK study found that one in five women reported menstrual disturbances post-vaccination, a rate considered above the threshold for a “very common” adverse reaction. Given these findings, clinicians must counsel women about the potential for menstrual changes after COVID-19 vaccination and advise them to seek medical attention if these changes are severe [12].

Consistent with general trends, most of our respondents contracted COVID-19. Notably, following the pandemic, there was an increase in the number of women diagnosed with premenstrual syndrome. Although this increase was possibly due to the physiological effects of the virus, it is necessary to consider the broader psychological context of the pandemic in terms of whether these menstrual patterns were influenced by stress arising from societal changes, pandemic-induced isolation, and academic pressure.

We observed an alteration in menstrual cycle regularity pre- and post-pandemic, with post-pandemic cycles showing more irregular behavior. This finding aligns with a study by King et al. [13], who reported that external stressors such as those resulting from a pandemic might trigger irregularities in menstruation. However, our findings differ from those of Dragun et al. [14], who reported no significant changes in menstrual patterns during stressful situations. It is important to investigate whether these discrepancies resulted from different responses of younger participants to stress or to other less well-known factors.

The potential effect of COVID-19 vaccination on menstrual cycle regularity is a new area of research. Some participants reported more disrupted cycles after vaccination. This finding is consistent with a study by Saadedine et al. [15], which suggested possible short-term changes in menstrual patterns after vaccination, hypothesized to be linked to immune responses. Such findings contrast with those of Moolamalla et al. [16], who noted minimal post-vaccination menstrual changes. Given that most of our respondents received all three vaccine doses, the possibility of a cumulative effect or individual variability should be considered. Furthermore, 89.9% of the respondents did not use hormonal contraceptives, and 87.1% reported no chronic illnesses. Given that contraceptives can significantly affect menstrual cycle regularity, the low usage rates are striking and may be influenced by specific social dynamics, age trends, or cultural patterns.

The findings of this study suggest potential associations between the COVID-19 pandemic, subsequent vaccinations, and changes in menstrual patterns among young female students and highlight the complexity of these interactions. Future research should be more comprehensive, involving diverse cohorts and longitudinal methodologies to understand better the underlying mechanisms and causal relationships involved. Greater awareness of the potential psychosocial and physical effects of the pandemic, as highlighted in a study by Alshammari et al. [17], could help health authorities optimize their preparedness for future pandemics. For example, health authorities in Saudi Arabia launched numerous free online applications and hotlines (937) for individuals to consult with health professionals during the pandemic, and such initiatives must be expanded.

Study limitations

The limitations of this study include its small sample size, with female students recruited from a single center only at KSU, and its cross-sectional design, which only captured a single point in time. Our findings may not reflect the experiences of the broader population, as most participants were medical undergraduates. The study’s cross-sectional design prevented exploring potentially significant changes in menstrual patterns over extended periods or shifts outside the study timeframe. In addition, recall bias may have affected the accuracy of the results, as participants were asked to recall their pre-pandemic conditions and menstrual irregularities. Finally, the absence of a control group, especially one not exposed to the same stress levels, makes it challenging to draw definitive connections between the observed menstrual changes and the pandemic or vaccination efforts.

## Conclusions

This study highlights potential connections between the COVID-19 pandemic, subsequent vaccinations, and changes in menstrual patterns, which can help clarify the pandemic’s physiological and psychological effects and the vaccination campaigns on women’s health. However, owing to the complex interplay of these factors, future studies should adopt a comprehensive approach and more extensive investigation that considers biological and psychosocial factors, lifestyle, and dietary habits in larger samples from multiple centers to clarify the underlying mechanisms and causal relationships.
